# Analysis of Relationship between Natural Standing Behavior of Elderly People and a Class of Standing Aids in a Living Space

**DOI:** 10.3390/s22031178

**Published:** 2022-02-04

**Authors:** Yusuke Miyazaki, Kei Hirano, Koji Kitamura, Yoshifumi Nishida

**Affiliations:** 1 Department of Systems and Control Engineering, Tokyo Institute of Technology, 2-12-1, O-okayama, Meguro-ku, Tokyo 152-8552, Japan; hirano.k.ai@m.titech.ac.jp; 2 Artificial Intelligence Research Center, National Institute of Advanced Industrial Science and Technology, 2-3-26, Aomi, Koto-ku, Tokyo 135-0064, Japan; k.kitamura@aist.go.jp (K.K.); nishida.y.af@m.titech.ac.jp (Y.N.); 3 Department of Mechanical Engineering, Tokyo Institute of Technology, 2-12-1, O-okayama, Meguro-ku, Tokyo 152-8552, Japan

**Keywords:** elderly behavior library, RGB-D camera, elderly standing behavior, safer environment

## Abstract

As the world’s population ages, technology-based support for the elderly is becoming increasingly important. This study analyzes the relationship between natural standing behavior measured in a living space of elderly people and the classes of standing aids, as well as the physical and cognitive abilities contributing to household fall injury prevention. In total, 24 elderly standing behaviors from chairs, sofas, and nursing beds recorded in an RGB-D elderly behavior library were analyzed. The differences in standing behavior were analyzed by focusing on intrinsic and common standing aid characteristics among various seat types, including armrests of chairs or sofas and nursing bed handrails. The standing behaviors were categorized into two types: behaviors while leaning the trunk forward without using an armrest as a standing aid and those without leaning the trunk forward by using an arrest or handrail as a standing aid. The standing behavior clusters were distributed in a two-dimensional map based on the seat type rather than the physical or cognitive abilities. Therefore, to reduce the risk of falling, it would be necessary to implement a seat type that the elderly can unconsciously and naturally use as a standing aid even with impaired physical and cognitive abilities.

## 1. Introduction

With the aging of the world’s population, technology-based support for the daily lives of the elderly has become an important issue. The WHO reports that between 2015 and 2050, the population of people aged 60 and older will increase from 12% to 22% [1]. Age-related changes in physical and cognitive abilities can lead to increased risks of falling [2,3]. In order to realize safer living environments and consumer products for the elderly, it is necessary to develop systems that are suitable for the daily consumer product use behavior of elderly people with declining physical and cognitive abilities. 

Smart homes and related ambient sensing technologies powered by machine learning (ML) and Internet of Things (IoT) devices are being proposed to provide living environments that support the elderly’s safety. The travel pattern of elderly people with dementia was categorized by ML model based on collected movement data by active RFID activity monitoring systems [4]. Machine learning models were applied to recognize the behavior and location of a participant using wearable sensors provided as a smartwatch [5]. Daily activity patterns of people with dementia were analyzed by ML methods based on data recorded via environmental sensors [6,7]. A classification model for dementia was developed by extracting and selecting distinctive features based on the dynamic ranking based on a public dataset of daily life activities of adults in a smart home [8]. Outcomes for older adults in wearables and artificial intelligence (AI)–powered digital health community was improved [9]. These studies show the usefulness of IoT and machine learning for measuring the daily activities of the elderly. However, these studies do not evaluate behaviors based on the measurement of whole-body behaviors in daily living spaces. If the whole-body motion of a human in daily living space can be analyzed, it will be possible to assess the physical burden of the elderly caused by the characteristics of their body motions, such as the risk of falling. Therefore, to analyze elderly people’s behavior and support in their daily lives, other studies have developed a marker-less motion capture system that utilized an RGB-D camera, such as Kinect. For example, a dual-task training system using Kinect was developed to improve the physical and cognitive abilities of the elderly [10]. A health monitoring system using Kinect was developed and walking, standing up, and sitting down into normal and unusual movements were categorized [11]. A framework based on fog computing was presented for convenient and efficient physiological function assessment based on joint mobility measures by using Kinect [12]. A total of 75 different gait samples were measured and analyzed by Kinect v2 with a real-time approach [13]. These studies enable a non-invasive and simple analysis of the whole-body behavior of the elderly in their daily living space. Furthermore, a dataset of daily activities of the elderly was developed, in which the daily activities of 50 elderly were measured with Kinect v2 and classified into 55 actions [14]. A large elderly behavioral dataset will contribute to improving the accuracy of elderly behavior recognition. However, from the viewpoint of consumer product safety for elderly people with degradation of physical and cognitive abilities, it is essential to focus more on the relationship between consumer product use behavior of the elderly and the design characteristics. 

Therefore, it is necessary to measure consumer product use behavior of elderly people in various daily living environments and to understand common characteristics in relation to cognitive and physical abilities. Analysis of consumer product use behavior of elderly people has generally been conducted in a laboratory environment [15,16,17]. However, in daily environments, when cognitive and physical abilities decline gradually, consumer products may be used in ways that designers do not intend. The consumer products used in daily environments by elderly people with changing cognitive and physical abilities are mainly consumer products made for generally healthy people or welfare equipment. In other words, there is a lack of consumer products that the elderly, who do not need even welfare equipment, can use naturally in their daily environment to enhance their safety. To solve these issues, it is necessary to quantitatively understand how elderly persons with various physical and cognitive abilities use consumer products in their actual daily environment, not in a controlled environment such as a laboratory environment.

Regarding consumer product use behavior measurement of elderly people in daily environments, we have developed an “elderly behavior library” [18,19,20]. The “elderly behavior library” includes RGB-D videos that recorded the natural behavior of elderly people when using consumer products placed in their residences or residential facilities. In addition, this library contains the physical ability index of the elderly based on the Barthel index (BI) [21] and the cognitive ability index based on the Mini-Mental State Exam (MMSE) [22] for each participant. To analyze the consumer product usage behavior recorded in the elderly behavior database, we developed a method comprising 3D-pose estimation during consumer–product interaction, normalizing standing behaviors that enable product-to-product comparisons, while clustering and visualizing the characteristics of consumers’ product use behaviors [23]. However, the relationships between consumer product use behavior, physical ability, and cognitive ability have not been quantitatively evaluated. In addition, how consumer products can change elderly behaviors and how elderly behavior relates to changes in cognitive and physical abilities in actual living environments has not been evaluated.

This research focuses on sit-to-stand behavior from chairs, sofas, and nursing beds recorded in the elderly behavior library because the sit-to-stand behavior of the elderly is related to the risk of falling [24,25]. Many biomechanical studies on standing behavior have been conducted in the laboratory environment [26,27,28,29,30]. In addition, a simpler method to measure the standing behavior using wearable sensors has been developed [31,32]. From the viewpoint of the relationship between the product design and standing behavior, analysis of the relationship between standing behavior and chair [15] or handrail [17] design have been conducted. However, it is difficult to reproduce the natural behavior of the elderly in a controlled laboratory environment. Furthermore, the relationship between standing aid usage and natural standing behavior based on ambient measurements in actual living environments has not been analyzed. 

If we can uniformly evaluate intrinsic and common standing aid characteristics among the various type of seats to support the behavior of an elderly person, we will be able to provide a “human behavior support-centered design” index, regardless of the type of seats. For example, armrests for chairs and sofas, handrails for nursing beds, and handrails for toilets and bathrooms are all essential classes of standing aids to support sit-to-stand behavior, even though they have different names, shapes, and configurations depending on the seat type. If countermeasures are taken by focusing only on the seat type itself, in which an injury occurs, it may be possible to prevent injuries to that product. However, similar injuries recur in other types of seats that are used in the same way. Therefore, it is necessary to develop and utilize a method that enables the evaluation of the intrinsic class of standing aids to support an elderly person in each type of seat normally, independent of the seat type. 

Therefore, the purpose of this study was to analyze the relationship between the standing behavior of the elderly in an actual living environment and an intrinsic class of “standing aids” of various seat types, cognitive ability, and physical abilities, especially from the data in the elderly behavior library. First, to analyze the relationship between standing behavior and the standing aid of a seat based on natural observations in daily life, we extracted 24 cases of standing behavior in a chair with armrests, a sofa with armrests, and a nursing bed with handrails from the elderly behavior library. In the analysis of standing behavior, we developed and utilized a method that enables the comparison and visualization of the relationship between standing behavior and intrinsic and common standing aid characteristics among the various types of seats. Finally, based on the results of cluster analysis and visualization of standing behavior in daily living environments, the relationship between the standing aid characteristics, cognitive ability, and physical abilities, and the natural standing behavior of elderly people in daily living space was investigated.

## 2. Materials and Methods

### 2.1. Standing Behavior Recorded in the Elderly Behavior Library

**Figure 1:**
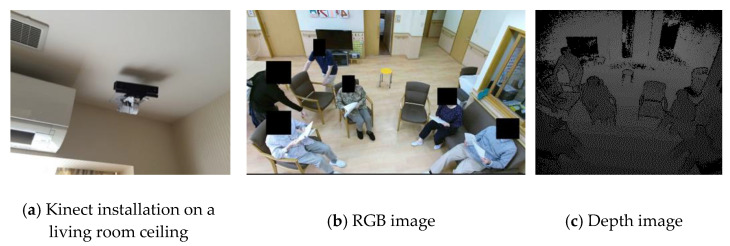
Example of installation of Kinect in a living room in a nursing home and captured RGB and depth image.

An elderly behavior RGB-D video library was previously constructed by the authors [18,19,20]. The videos recorded in the library illustrate how different consumer products (beds, chairs, wheelchairs, canes, doors, kitchen tools, and handrails) are utilized by the elderly, depending on age, but also on cognitive ability, physical ability, and the level of care needed. Microsoft’s Kinect v2 was installed in daily environments such as nursing homes and ordinary homes (N = 43, 52 to 104 years old). As shown in Figure 1a, the Kinect was placed near the ceiling of a living room and a corridor in a nursing home, to avoid motion occlusion as much as possible. The Kinect v2 enables RGB video, depth images, and skeleton sequence (Figure 1b,c). The resolution of the RGB video was 1920 × 1080, and the depth map was 512 × 424. The database comprises 1,211 situation videos. Situation videos include places (cafeteria, living room, kitchen, stairs, gateway, prayer room, etc.) and products (table, chair, sofa, a bed, shelf, laundry appliance, kitchenware, handrail, cane, home shrine, etc.). 

This study analyzed consumer product use behavior in various standing behaviors recorded in an elderly behavior library. In this study, we analyzed 24 cases of standing behaviors of six participants from the elderly behavior library, which narrowed down to all standing behaviors from a sofa, a chair, and a nursing bed, and whose whole-body postures were successfully extracted. Table 1 lists the sex and age of the participants, physical ability index of the elderly based on the BI, cognitive ability index based on the MMSE (Appendix A), and seat type utilized by the participants during the standing behavior. It is worth noting that the ID in Table 1 is similar to that recorded in the elderly behavior library. 

**Figure 2:**
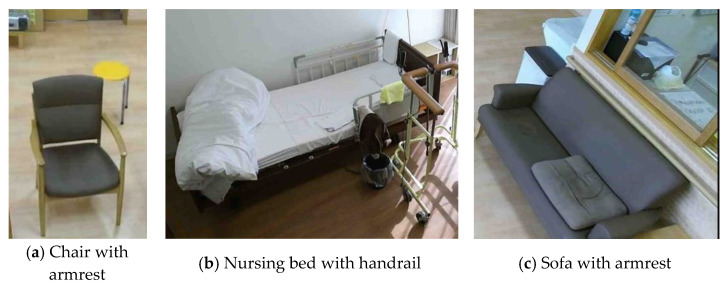
The products analyzed for standing behavior.

Three types of seats were used in the standing behaviors: a nursing bed with a handrail, a chair with an armrest, and a sofa with an armrest, as illustrated in Figure 2. These seats were originally installed in the nursing homes where the participants lived, and the measured standing behaviors were observed in a situation where the participants used them naturally daily. All participants gave their informed consent for inclusion before they participated in the study. The study was conducted in accordance with the Declaration of Helsinki, and the protocol was approved by the Tokyo Institute of Technology Human Subjects Research Ethics Review Committee (Protocol Code 2019150) and the AIST Ergonomics Experiment Review Committee (Protocol Code 2016-659H).

### 2.2. Method for Analyzing Product Use Behavior Stored in the Elderly Behavior Library

**Figure 3:**
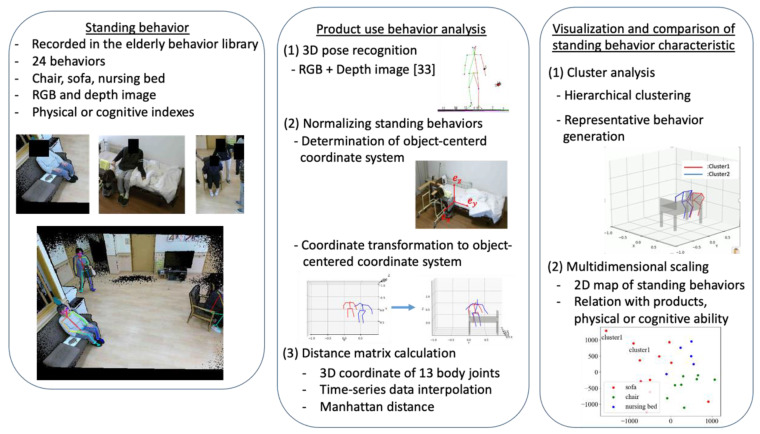
Outline of proposed method.

If we can uniformly evaluate the intrinsic class of the standing aid of a seat to support elderly behavior, we will be able to give a “human behavior support-centered” design index regardless of the product. Therefore, it is necessary to develop and utilize a method that enables the evaluation of the “class of a standing aid”, which is intrinsic to each type of aid in a common manner, independent of the product. For this purpose, it is necessary to develop and utilize a method that can normalize and compare the standing behavior captured in different environments with different camera configurations and with different seats. In analyzing the natural standing behavior of the elderly recorded in the elderly behavior library, we utilized a method developed to normalize and compare the human behavior among different products [23]. Figure 3 shows an outline of our method. The method comprises (1) 3D-pose estimation during consumer product usage [33], (2) normalizing standing behaviors that enable product-to-product comparisons, (3) while clustering and visualizing the characteristics of standing behaviors.

It is necessary to compare the standing behavior under the condition that different types of seats are used and the cameras are installed in different locations. Therefore, we defined a coordinate system based on the purpose of the standing aid use and named it the “object-centered coordinate system”. Regarding a product evaluation focusing on the class of standing aid, elbow rest of sofa and chair or handrails of a nursing bed are the main evaluation targets. Therefore, in this study, the origin of the coordinate system was set to a point at the base of the armrest or handrail. First, the 3D coordinates of the point clouds of the surfaces of armrest or handrails of chairs, sofas, and beds, **P**, were obtained from the depth data. $P={\left[\begin{array}{ccc} X & Y & Z \end{array}\right]}^{T}$ is the 3D coordinate of the seat surface measured in the camera coordinate system. Then, the point at the base of the armrest or handrail was set as the origin $P_{0}$ of the coordinate system. The next step is to set up an orthonormal basis from the 3D coordinate point cloud of the seat. First, the parameters *a*, *b*, *c*, and *d* of a plane Equation (1), representing the seat surface from the 3D point cloud, were determined. As the surface of the camera is not always perpendicular to the camera, *c* = 1 was assumed.

(1) $$\left[\begin{array}{c} X\\ Y\\ 1 \end{array}\right]\left[\begin{array}{c} a\\ b\\ d \end{array}\right]=-cZ$$

Solving Equation (1) using the least-squares method returns Equation (2), where $P^{\prime }={\left[\begin{array}{ccc} X & Y & 1 \end{array}\right]}^{T}$.

(2) $$\left[\begin{array}{c} a\\ b\\ d \end{array}\right]=-{\left({P^{\prime }}^{T}P^{\prime }\right)}^{-1}{P^{\prime }}^{T}Z$$

Next, the normal vector was obtained from the 3D coordinate point cloud of each seating surface. Using *a* and *b* calculated in Equation (2), the normal vector of the seating surface, $e_{z}$, is defined as in Equation (3).

(3) $$e_{z}={\left[\begin{array}{ccc} a & b & c \end{array}\right]}^{T}$$

and this was set as the vertical basis vector $e_{z}$.

Further, ${e_{x}}^{\prime }$ in the front–back direction was calculated from the 3D point cloud of the backrest in the case of chairs and sofas, and the side frame in the case of nursing beds, using the same method as calculation of $e_{z}$.

In addition, the basis vector $e_{y}$ in the left and right directions were calculated using the cross product.

(4) $$e_{y}=e_{z}\times {e_{x}}^{\prime }$$

As ${e_{x}}^{\prime }$ is not orthogonal to $e_{z}$ and $e_{y}$, the true basis vector $e_{x}$ was recalculated from $e_{z}$ and $e_{y}$ by the cross product.

(5) $$e_{x}=e_{y}\times e_{z}$$

The orthonormal basis of the object-centered coordinate system was then used to define the rotational matrix **R**.

(6) $$R={\left[\begin{array}{ccc} e_{x}{}^{T} & e_{y}{}^{T} & e_{z}{}^{T} \end{array}\right]}^{T}$$

Finally, orthonormal transformation matrix H for each trial was calculated, which was subsequently utilized to perform the coordinate transformation of the standing aid usage behavior.

(7) $$H=\left[\begin{array}{cc} R & P_{0}\\ 0 & 1 \end{array}\right]$$

**Figure 4:**
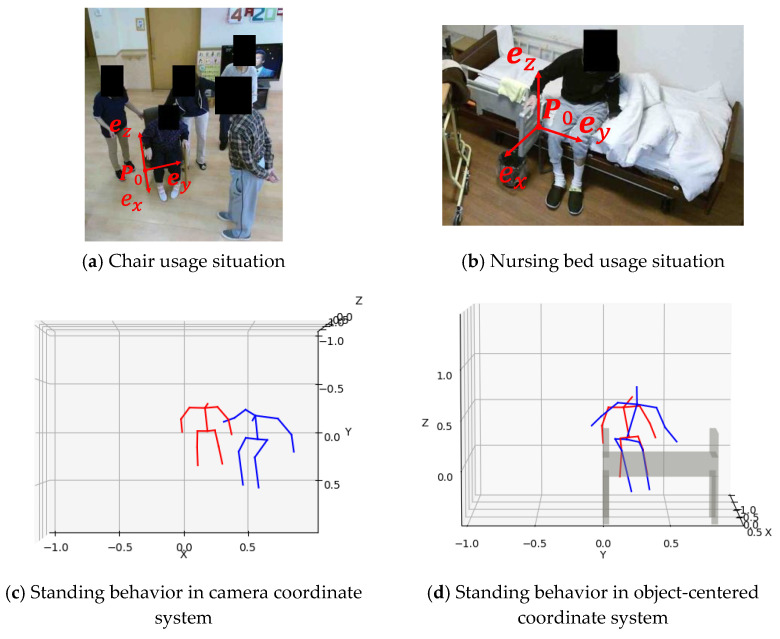
Comparison of two elderly persons standing behavior expressed in camera coordinate system and object-centered coordinate systems: red, chair usage case; blue, bed usage situation.

As an example of these processes, Figure 4 illustrates a comparison of the standing behavior from a chair and nursing bed before and after the coordinate transformation to the object-centered coordinate system. Figure 4c illustrates the posture of the participant compared to the camera coordinate system. Figure 4d illustrates the posture of the participant compared to the object-centered coordinate system. The original camera coordinate system is represented by a completely different standing behavior. However, by unifying motions using the object-centered coordinate system, the standing behavior in the case of a chair can be compared with the standing behavior in the case of a nursing bed.

Normalized standing behaviors that enable product-to-product comparisons were analyzed based on cluster analysis. As the number of clusters was not known in advance, and the number of cases was relatively small, i.e., 24, hierarchical cluster analysis was used as the cluster analysis method. The unweighted pair group method using the arithmetic average (UPGMA) [34] and Ward’s method [35] were used as linkage methods. The features used in the cluster analysis are the time series of the 3D coordinates of 13 body joints: left shoulder, left elbow, left wrist, right shoulder, right elbow, right wrist, center pelvis, left pelvis, left knee, left ankle, right pelvis, right knee, and right ankle. The distance matrix $D_{sum}$ is the sum of the Manhattan distance between the trajectories of the 3D coordinates of each joint and each case, as described next.

The 3D coordinate data for each marker is a time-series dataset from time steps 1 to T. First, the time-series data of each standing behavior were interpolated by a cubic spline function, and the time-series data of each action was unified to 500 frames. From a 3D joint coordinate data for each participant, the distance matrix $D_{j}$ that represents the difference in the marker coordinate trajectories between all 24 standing behaviors was calculated. $D_{j}\left(K,L\right)$, the element of $D_{j}$ that represents the difference in the trajectory of joint *j* between the *K*-th standing behavior ($J_{k}$) and the *L*-th standing behavior ($J_{L}$), is expressed by Equation (8).

(8) $$D_{j}\left(K,L\right)=\overset{500}{\underset{t=1}{\sum }}\left|J_{Kt}-J_{Lt}\right|$$

As the number of observed joint markers is 13, the distance matrix $D_{sum}$ representing the standing behavior dissimilarity between all standing behaviors is expressed by Equation (9).

(9) $$D_{sum}=\overset{13}{\underset{j=1}{\sum }}D_{j}$$

Finally, we performed hierarchical clustering of all motions using a distance matrix $D_{sum}$. The number of clusters is determined based on the silhouette coefficient [36] and dendrogram.

The next step is to create cluster average behavior that represents each cluster. Suppose that *n* kinds of behaviors are clustered in a cluster of interest. Then, assuming that a single behavior is represented by a multidimensional matrix $X_{i}$, the average behavior $X_{ave}$ is represented by Equation (10).

(10) $$X_{ave}=\frac{1}{n}\overset{n}{\underset{i=1}{\sum }}X_{i}$$

In addition, two-dimensional maps were created by the multidimensional scaling method using the distance matrix described in Equation (9). The multidimensional scaling method is a method for calculating a low-dimensional vector $X_{i}$ that minimizes the objective function defined by Equation (11). The program code was written in C++ and Python, with scipy used for cluster analysis and Scikit learn for MDS.

(11) $$\mathrm{argmin}\left(\overset{n}{\underset{i<j}{\sum }}{\left(d_{ij}-z_{i}-z_{j}\right)}^{2}\right)$$

## 3. Results

### 3.1. Cluster Analysis and the Representative Behaviors of Elderly Standing Behavior

**Figure 5:**
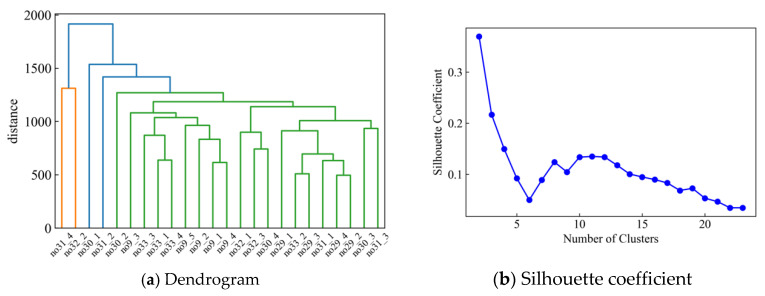
Clustering result of 24 standing behavior from chair, sofa, and nursing bed.

**Figure A1:**
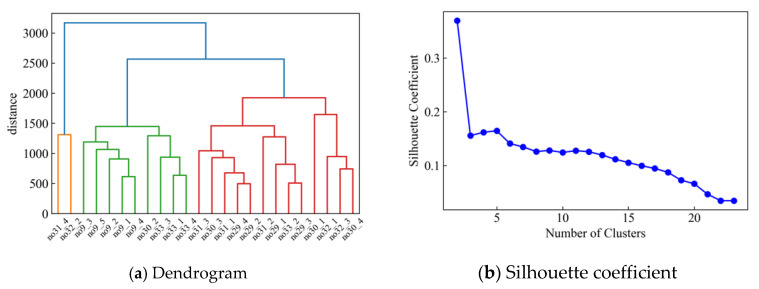
Clustering results of 24 standing behaviors from chair, sofa, and nursing bed using Ward’s method.

The dendrogram and silhouette coefficients formed by cluster analysis using UPGMA are illustrated in Figure 5. In the dendrogram, the largest vertical distance that does not intersect any of the other clusters is between clusters 2 and 3. Regarding the silhouette coefficient, the maximum value is obtained when the number of clusters is two. After that, the score decreases with an increase in the number of clusters, increases again when the number of clusters reaches 7, and reaches the maximum value again when the number of clusters is approximately 11. As the silhouette coefficient score is the largest when the number of clusters is two, the optimal number of clusters for this analysis was two. Cluster 1 is no31_4 and no32_2, and Cluster 2 includes the other samples. The same results were obtained through clustering using Ward’s method (Figure A1a,b, Appendix B).

**Figure 6:**
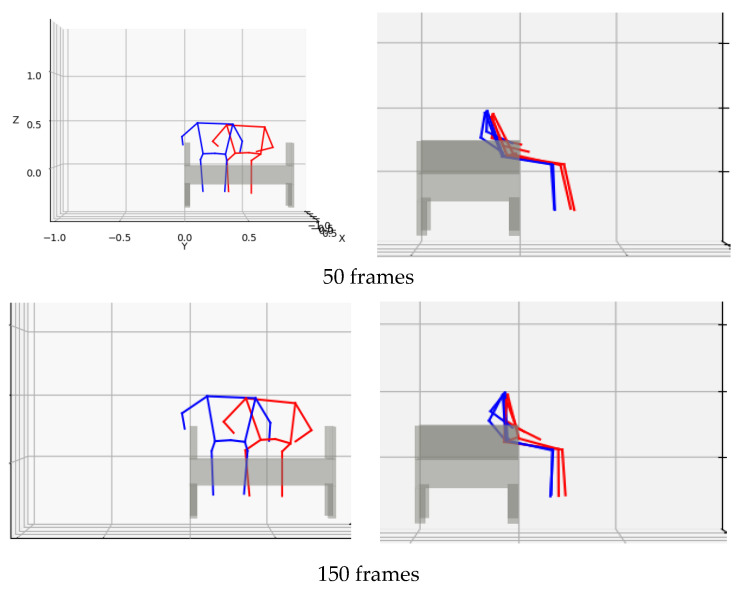

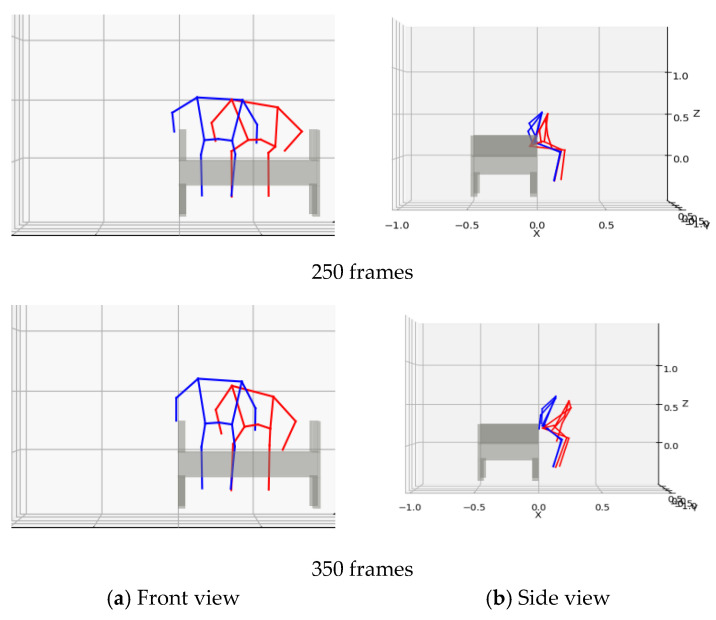
Average standing behavior in each cluster: red: cluster 1; blue: cluster 2.

Figure 6 illustrates the average motion of each cluster in an object-centered coordinate system. The red and blue stick figures show the representative behavior of cluster 1 and cluster 2, respectively. From the front view, cluster 1′s motion shifted farther away from the elbow rest. From the side view, the left and right shoulders of cluster 1′s motion moved forward by approximately 250 frames, compared with cluster 2′s motion. The posture of cluster 1 at the end of the standing behavior was tilted forward, compared with that of cluster 2. When we checked the actual actions categorized as cluster 1, we determined that one of the actions was standing up with hands on the elbow rest and seat, and the other was sitting near the center of the sofa and standing up with hands on both knees. In addition, the front view of Figure 4 illustrates that cluster 2 primarily includes standing up in a position close to the elbow rest. When we checked the behavior of cluster 2 in all cases, 20 out of 22 cases used the elbow rest.

### 3.2. Relationship between Standing Behavior and Product Use

**Figure 7:**
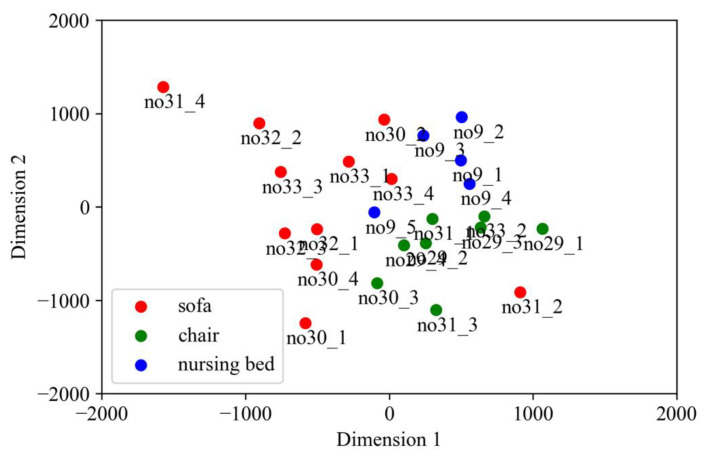
Standing behavior map categorized by products used.

Figure 7 illustrates a two-dimensional map created by the multidimensional scale construction, based on the products used in each standing behavior. Both no31_4 and no32_2 in cluster 1 used the sofa. The behaviors using the sofa, except for no31_2, were concentrated in the upper left direction of the 2D map. The behaviors using a nursing bed were placed in the center of the right side, and the standing behaviors using a chair were placed on the lower right side.

### 3.3. Relationship between Standing Behavior and Physical Ability and Cognitive Ability

**Figure 8:**
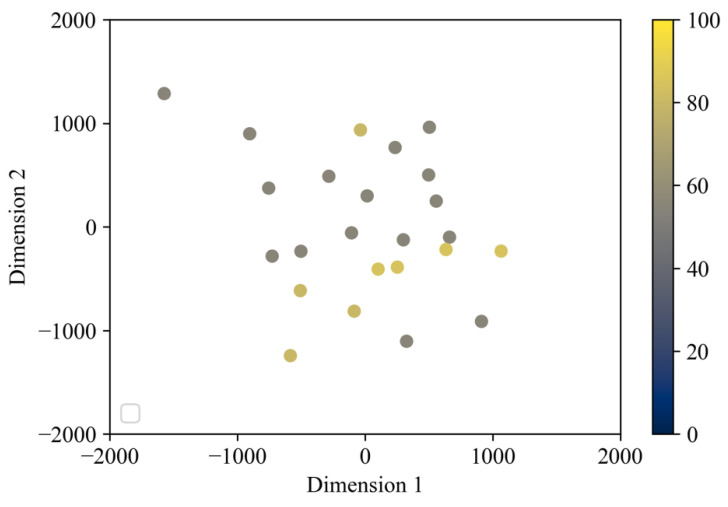
Standing behavior map categorized by BI.

**Figure 9:**
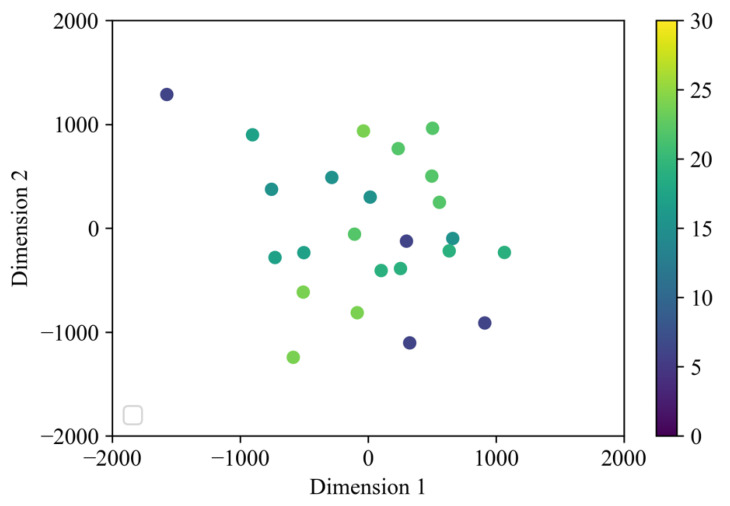
Standing behavior map categorized by MMSE.

Figure 8 illustrates a two-dimensional map, colored based on BI, a physical ability index. In BI, participants with a score of 60 or lower require assistance mainly for standing and sitting, and participants with a score of 60 or lower experience weakness in the muscles of the legs, waist, and trunk; however, standing cases with a score of 55 points were evenly distributed on the map. Figure 9 illustrates a two-dimensional map colored using the MMSE, an index of cognitive ability. Participants with low cognitive ability scores (no31) were distributed evenly from the lower left to the lower right. The motions of participants with relatively high scores were also widely distributed from the lower left to the upper right of the map.

## 4. Discussion

### 4.1. Cluster Analysis and the Representative Standing Behaviors

This is the first study to analyze the relationship between natural standing behavior measured in the living space of the elderly and products, physical abilities, and cognitive abilities that contribute to the prevention of fall injuries in households. Previous biomechanical research has been conducted in a laboratory environment where participant behavior is limited [15,16,17,25,26,27,28,29,31,37,38,39]. Therefore, the relationship between natural daily standing behavior and products has not yet been analyzed. In addition, several behavioral databases for the elderly that store images have been developed in recent years [14,40,41]. However, these studies have focused on labeling the behavior itself and have not labeled the four relationships among elderly behavior, products, cognitive abilities, and physical abilities that are important for injury prevention. Furthermore, this study utilized a method for normalizing, clustering, and visualizing behaviors among different types of seats to evaluate the intrinsic class of standing aid that is common among various seats used in living spaces. This method enables us to evaluate the difference in standing behavior by focusing on the intrinsic and common class of standing aids of various seats, which may contribute to the improved safety of the lives of the elderly.

The standing behaviors were divided into two major clusters: cluster 1 was the standing behavior without using armrests or handrails, and cluster 2 was the behavior of using the standing aid of these seats (armrests or handrails). The center of gravity was kept within the base of the support in the standing behavior. In cluster 1, the base of the support was considered to be narrow in the anterior–posterior direction, because the elbow rest was not used effectively near the point where the trunk began to rise at approximately 250 frames in the middle of the standing period. Therefore, the trunk was tilted forward, and the center of gravity might be moved forward in the anterior–posterior direction. 

In contrast, in cluster 2, the base of the support was wider in the posterior direction because the projected position of the hand on the elbow rest was added to the base of the support. Therefore, it was interpreted as a cluster 2 behavior that can support the body, even if the upper body was not tilted forward in the middle of standing, and can smoothly shift the body’s center of gravity throughout the standing behavior.

From the above points, the behavior of cluster 1 cannot effectively utilize the armrests or handrails, which are the standing aid of the seats, resulting in a shift in the center of gravity, which increases the risk of falling [27].

However, cluster 2 behavior is considered to be a motion that effectively utilizes the standing aid of the seats and reduces the risk of falling when standing.

### 4.2. Relationship between Product Use and Representative Standing Behaviors of Each Cluster

As illustrated in Figure 7, the upper left corner of the map indicates the tendency of cluster 1 behavior. However, the lower right corner of the map is considered to have a stronger tendency for cluster 2. 

The chair, which is the seat concentrated in the lower right corner of the map, is equipped with armrests on both sides of the body; hence, the position of the body is limited to the vicinity of the armrests. Therefore, the behavior of cluster 2 is considered to be dominant in the case of chairs.

The sofa cases tend to be concentrated in the upper left corner of the map. As sofas have a wider seat surface than chairs and there are no restrictions on the sitting position, the elderly may sit far from the armrest. Therefore, in the case of a sofa, the behavior of cluster 1, which is to stand up without using the armrest, is dominant. Although case no31_2 is the only example of a sofa case that was not placed in the upper left corner, this is because the elderly user was able to stand up using the armrest in this case.

Nursing beds tend to be placed at the center of the map. As the nursing bed has a relatively wide seat, the user can easily sit in a free position against the handrail. Therefore, the user may not use the handrail when standing up. However, there are cases in which the handrail is used to raise the upper body when the user wakes up from a lying position, and then, the handrail is used to raise the body when the user moves to a standing position. Therefore, the nursing bed placed in the center of the map is considered an intermediate case between the chair and sofa.

### 4.3. Relationship between Standing Behavior and Physical or Cognitive Ability Scores 

Regardless of the scores of the physical and cognitive abilities, these standing behaviors were scattered evenly on the standing behavior map. Specifically, regardless of the change in physical and cognitive abilities, the position of the two-dimensional map of standing behavior was determined by the product used. This suggests that even for elderly people whose physical and cognitive abilities have declined, the risk of falling can be reduced by placing seats with a high standing aid capability in the living environment and by using these seats naturally. However, when seat types with a wide seat surface, such as sofas, were placed in the living environment, the elderly user tended to perform a standing behavior with a higher risk of falling, as indicated in cluster 1. Therefore, to improve the living space and reduce the risk of falling, it will be necessary to avoid placing products with a wide seat surface, such as sofas, in which the armrests cannot be used naturally, and to design and introduce products that can control the sitting position of users so that they can unconsciously and naturally use the standing aid from a sitting position.

### 4.4. Limitations and Future Scope

The number of subjects was 6, and the number of trials was 24; therefore, in the future, we need to increase the number of trials conducted. In addition, since the database is based on the natural observation of the behavior of the participants in their daily lives without any instructions or restrictions, the dataset will be biased in terms of what product is used by the participants. Despite the above problems, this study utilized the elderly behavior library because it has the advantage of being annotated products, elderly behaviors, cognitive abilities, and physical abilities, all of which are important in preventing injuries among the elderly in daily life. To increase the number of trials, it will be necessary to utilize other databases of elderly behavior [14,40] by adding annotations with products, cognitive abilities, and physical abilities. If the number of trials can be increased, it will be possible to analyze the relationship between more detailed product design parameters, such as the dimensions or materials, and the standing behavior, or to analyze whether the standing ability of the elderly differs depending on their physical and cognitive abilities when the products have the same product characteristics. 

In this study, we conducted a hierarchical cluster analysis using the Manhattan distance of the joint trajectories between standing behaviors. As a result, we found that the standing behavior was categorized according to the product, and we were able to examine the relationship between standing behavior and products, body, and cognitive ability in a two-dimensional map constructed by MDS. However, if the number of trials increases, cluster analysis for large-scale data, such as non-hierarchical cluster analysis, can be considered. In addition, it will be necessary to consider several other candidates for the similarity matrix of time series data of joint coordinates, such as the DTW distance.

It is also necessary to conduct a detailed biomechanical analysis using a force plate system or a more accurate motion capture system to quantitatively evaluate the relationship between standing behavior and product characteristics, cognitive abilities, and physical abilities. To accomplish this, there are two options—introducing a force plate and a more accurate marker-less motion capture system in the daily life environment or experimenting in a laboratory environment with a sophisticated measurement system. For the former, it is necessary to develop and introduce a simple and inexpensive force plate system that can be embedded in the living space. For the latter, it is difficult to reproduce the natural behavior of the elderly in the controlled space of the laboratory environment. Therefore, it would be necessary to perform research by first capturing the actual usage behavior of the elderly in the daily life environment and then conducting detailed analysis in a laboratory environment with better measurement equipment.

## 5. Conclusions

The main contributions of this study are as follows: (1) We analyzed, for the first time, the relationship between the natural standing behavior of elderly people and the intrinsic and common class of standing aids of sofas, chairs, and nursing beds, where natural standing behaviors occur in a living space. (2) We showed that the characteristics of the standing aid of a chair, sofa, and nursing bed are more related to safer standing behaviors than cognitive and physical abilities. Finally, (3) we demonstrated that the standing behaviors can be categorized into safe and riskier standing behaviors depending on the design characteristics of the standing aids of chairs, sofas, and nursing beds. 

First, we developed a method for normalizing, clustering, and visualizing standing behaviors among different products. This method enables us to evaluate the difference in standing behavior by focusing on the intrinsic and common standing aid among the different types of seats, which may contribute to improved safety of the lives of the elderly. 

The results showed that the standing behaviors of the elderly were categorized into two clusters: 2 standing behaviors while leaning forward without using an armrest of the sofa as a standing aid, and 22 behaviors without leaning forward by using an armrest of the sofa, or chair, and a handrail of nursing bed as a standing aid. In addition, standing behaviors were categorized according to the type of seat, regardless of physical or cognitive abilities. In particular, when a seat with a wide seat surface such as a sofa was placed in the living environment, the elderly tended to perform a standing behavior with a higher risk of falling because there were no restrictions on the sitting position, and cases occurred where the person sits far away from the armrest. Therefore, to improve the living space and reduce the risk of falling, it will be necessary to design and implement a seat that can control the sitting position of the elderly user so that the user can unconsciously and naturally use a class of standing aid of seats even when the elderly have impaired physical and cognitive abilities.

## Figures and Tables

**Table 1:** Motion ID and life ability score and product.

ID	Sex	BI	MMSE	Product
no9_1	M	55	22	Nursing bed
no9_2	M	55	22	Nursing bed
no9_3	M	55	22	Nursing bed
no9_4	M	55	22	Nursing bed
no9_5	M	55	22	Nursing bed
no29_1	F	85	19	Chair
no29_2	F	85	19	Chair
no29_3	F	85	19	Chair
no29_4	F	85	19	Chair
no30_1	M	80	24	Sofa
no30_2	M	80	24	Sofa
no30_3	M	80	24	Chair
no30_4	M	80	24	Sofa
no31_1	F	55	6	Chair
no31_2	F	55	6	Sofa
no31_3	F	55	6	Chair
no31_4	F	55	6	Sofa
no32_1	M	55	17	Sofa
no32_2	M	55	17	Sofa
no32_3	M	55	17	Sofa
no33_1	F	55	15	Sofa
no33_2	F	55	15	Chair
no33_3	F	55	15	Sofa
no33_4	F	55	15	Sofa

## Data Availability

The standing behaviors of elderly persons analyzed in this study can be accessed on the “elderly behavior library”. https://www.behavior-library-meti.com/behaviorLib/homes/about (accessed on 29 October 2021).

## Appendix A

BI is a scoring technique that measures performance in 10 activities of daily life [21]. The items can be divided into a group related to self-care and a group related to mobility. In Japan, a score of 100 on the BI score indicates independence, a score of 85 or lower indicates little assistance, a score of 60 or lower indicates assistance is required mainly for activities of daily living, and a score of 40 or lower indicates that assistance is required for almost all activities.

The MMSE is a 30-point test of cognitive functioning consisting of 11 items: time awareness, place awareness, an immediate and delayed replay of three words, calculation, object calling, sentence recitation, three-step verbal commands, writing commands, writing sentences, and graphic copying [22]. An MMSE score of 23 or below is indicative of dementia, while a score of 27 or below is indicative of mild cognitive impairment (MCI).
